# Between Guidelines and Daily Practice: The Persistent Use of Parenteral Depot Corticosteroids in Germany

**DOI:** 10.1002/clt2.70187

**Published:** 2026-07-09

**Authors:** Christian Taube, Timm Greulich, Sebastian Böing, Oliver Pfaar, Martin Wagenmann, Sven Becker, Mathias Sulk, Niklas J. Gerkau, Jan Claudius Schwitalla, Tim Vierbuchen

**Affiliations:** ^1^ Department of Pulmonary Medicine University Hospital Essen—Ruhrlandklinik Essen Germany; ^2^ Department of Respiratory, Intensive Care, and Sleep Medicine Marburg University Member of the German Centre for Lung Research (DZL) Marburg Germany; ^3^ PneumoPraxis Marburg Marburg Germany; ^4^ Schwerpunktpraxis Lungen‐ und Bronchialheilkunde, Allergologie und Schlafmedizin Meerbusch Germany; ^5^ Section of Rhinology and Allergy Department of Otorhinolaryngology, Head and Neck Surgery University Hospital Marburg Philipps‐Universität Marburg Marburg Germany; ^6^ Department of Otorhinolaryngology Düsseldorf University Hospital (UKD) Düsseldorf Germany; ^7^ Department of Otorhinolaryngology, Head and Neck Surgery University Hospital Tübingen Tübingen Germany; ^8^ Department of Dermatology University Hospital Münster Münster Germany; ^9^ Respiratory and Immunology, BioPharmaceuticals Medical AstraZeneca GmbH Hamburg Germany

**Keywords:** allergic rhinitis, asthma, COPD, Germany, parenteral depot corticosteroids

## Abstract

**Background:**

Parenteral depot corticosteroids (PDCs) continue to be prescribed for allergic and respiratory diseases, despite clear guideline recommendations against their use. This clinical practice raises significant safety concerns. We analyzed current PDC use in Germany and characterized prescribing patterns.

**Methods:**

Retrospective analysis of anonymized, longitudinal prescription data (IQVIA LRx) and evaluation of anonymized electronic medical record (EMR) data (IQVIA Disease Analyzer) from October 2023 to September 2024. Data were stratified by diagnosis, prescription frequency, prescriber specialty, region, age group and comorbidities.

**Results:**

The analysis of prescription data revealed that 9984 asthma and 5279 chronic obstructive pulmonary disease (COPD) patients received at least one PDC injection annually by general practitioners, which are the predominant prescribers. Of these patients, the majority received a single prescription. Regional differences were observed with particularly high prescription rates in western regions such as Rhineland‐Palatinate and Saarland. Monthly PDC prescription rates follow seasonal patterns of most common pollen allergens. Furthermore, over 23,000 patients with rhinopathy but without asthma or COPD received at least one prescription as well as approximately 3700 patients with chronic sinusitis with or without nasal polyps but no asthma or COPD.

**Conclusion:**

PDCs continue to be administered to a relevant number of patients with asthma, COPD, and upper airway diseases despite clear guideline recommendations against systemic steroid treatment. The injection frequency, as well as the association with allergic comorbidities and seasonal patterns are compatible with the treatment of seasonal allergies. This underscores the need for targeted educational initiatives and stronger implementation of guideline‐adherent therapies.

**Abbreviations**
AIT: allergen immunotherapy
ARIA: allergic rhinitis and its impact on asthma
ASHIP: association of SHI physicians
COPD: chronic obstructive pulmonary disease
CS: chronic sinusitis
DMAADs: disease‐modifying anti‐asthmatic drugs
EMR: electronic medical records
ENT: ear, nose, and throat specialist (otolaryngologist)
GINA: global initiative for asthma
GOLD: global initiative for chronic obstructive lung disease
GP: general practitioner
LRx: longitudinal prescription
NP: nasal polyps
OCS: oral corticosteroids
PDC: parenteral depot corticosteroids
PHI: private health insurance
Pulm: pulmonologist
RP: (vasomotor and allergic) rhinopathy
Rx: prescription
SHI: statutory health insurance

## 1 Introduction

The use of parenteral depot corticosteroids (PDCs) in the management of allergic and respiratory diseases such as allergic rhinitis, chronic rhinosinusitis, and asthma, remains a subject of ongoing debate in routine care. Historically, PDCs were introduced as an efficient strategy for rapid and sustained symptom control, particularly during severe exacerbations, severe allergic symptoms or treatment‐resistant cases. While a single PDC injection is perceived as convenient and may provide symptom relief for 4 weeks or longer, there is substantial evidence for serious adverse effects and long‐term risks associated with the use of PDCs. Similar to the use of other systemic steroids the risks include diabetes, adrenal suppression or osteoporosis [1, 2] as well as reports of tissue atrophy [3, 4, 5] and hip necrosis [6].

Given these risks and the availability of modern and effective anti‐allergic therapies such as second‐generation antihistamines, topical corticosteroids, allergen immunotherapy (AIT), and biologics [7], the use of PDCs for treatment of allergic diseases is categorically discouraged by guidelines such as the Allergic Rhinitis and its Impact on Asthma (ARIA) guideline [8] or the German AIT guideline [9]. While current clinical practice guidelines do not oppose the use of systemic steroids per se, they recommend a rational, cautious, and indication‐specific use, restricted to well‐defined clinical situations. According to an EAACI position paper from 2020, the short‐term use of systemic steroids in allergic rhinitis should never be considered as a first‐line therapy and is only indicated in cases where the patients are intolerant or do not respond to other treatments [10]. In line with national and international asthma guidelines, even systemic oral corticosteroids (OCS) should be reserved for acute exacerbations and used as maintenance therapy only under well‐defined circumstances, specifically when other treatment options, such as biologics, have failed to restore symptom control or are not indicated [11, 12]. In chronic obstructive pulmonary disease (COPD), the Global Initiative for Chronic Obstructive Lung Disease (GOLD) recommends cautious and time‐limited OCS use in exacerbations with no role for depot injections in routine care [13].

While adverse effects of OCS—especially in long‐term use—are well‐documented [14], the available data on PDCs lack robust evidence and no new studies have been conducted [15]. Thus, there is a high level of uncertainty about the long‐term effects of PDCs in comparison to OCS, especially if taken repeatedly. Furthermore, PDCs are depot injections that release corticosteroids over the course of several weeks [16], which means that if adverse events occur, the drug exposure cannot be reduced or stopped.

Although PDCs were introduced decades ago, there is limited evidence available of how widely PDCs are currently used in Germany. Despite strong and long‐standing discouragement of PDC use for allergic and airway diseases, some PDC preparations remain approved for treatment of seasonal allergic rhinitis. This study analyzes anonymized longitudinal prescription data (LRx) and electronic medical records (EMR) from October 2023 to September 2024 to better understand PDC utilization and to characterize patient populations by administration frequency, age groups, and comorbidities, as well as regional differences in PDC use.

## 2 Methods

### 2.1 Study Design and Data Sources

This retrospective, observational study utilized two complementary German healthcare databases to provide comprehensive insights into PDC prescribing patterns. Both data sources, the longitudinal prescription data (LRx) and the electronic medical records (EMR), are anonymized and were aggregated for reporting. No hypothesis testing was planned; the study was descriptive by design.

### 2.2 Longitudinal Prescription Data (LRx)

German prescription data were obtained from the IQVIA LRx database [17, 18]. IQVIA LRx is a comprehensive, anonymized prescription database capturing approximately 80% of all prescriptions dispensed through retail pharmacies nationwide and reimbursed by statutory health insurance (SHI; German: *Gesetzliche Krankenversicherung*), which covers roughly 90% of the German population. For this analysis, IQVIA LRx data covering the period from October 2023 to September 2024 were utilized. The database contains information for each prescription including dosage form and package (identified by *Pharmazentralnummer*), prescription date, and prescriber specialty as well as patients' age and sex (inferred on a first‐name basis). The data were further stratified by age group and the practice location at the Association of SHI Physicians (ASHIP; German: *Kassenärztliche Vereinigung*, KV; 17 regions) level.

Since LRx data lacks information on diagnosis, a machine learning model was used to assign patients to the most likely diagnosis. This approach was used and validated in several published pharmacoepidemiologic studies [19, 20, 21]. The model discriminates between asthma, COPD and other respiratory diseases based on prescription history as well as patient‐ and prescriber‐related information. The model demonstrates 70.96% precision and 70.52% specificity. As it predicts a single diagnosis, patients cannot be categorized as having both asthma and COPD. The study population was aligned with national estimates to account for incomplete coverage, using region‐specific projection factors to extrapolate case numbers to Germany's SHI‐ and private health insurance‐ (PHI‐) insured population [20, 21].

### 2.3 Electronic Medical Records (EMR)

Anonymized EMR data were obtained from the IQVIA Disease Analyzer database [22]. This database includes a representative panel of more than 2800 practices and more than 3500 physicians with a total of 17 million patients in the past 3 years and more than 30 years of history. This study is limited to data obtained from general practitioners (GP), pulmonologist and ear, nose, and throat specialists (ENT) practices (Table 1). Data from October 2023 to September 2024 were analyzed. Only physicians with continuous data delivery during the study time period and only patients with confirmed diagnosis (ICD‐10) of COPD (J44), asthma (J45), vasomotor and allergic rhinopathy (J30), chronic sinusitis (J32) or nasal polyps (J33) were included. Patients included in the study were further stratified by diseases of the musculoskeletal system and connective tissue (ICD‐10 M00‐M99). Here, subclassification of ICD‐10 M05‐M13 [Inflammatory polyarthropathies (excl. arthropathies in diseases classified elsewhere)], M15‐M19 (Osteoarthritis), and M70‐M79 (Other diseases of the soft tissue) have been applied. Patient numbers in the main figures were projected to match the total number of SHI and PHI insured patients in Germany with projection factors specific to each specialty (Table 1). No personal data were used; only anonymous patient data were analyzed and aggregated for reporting.

**TABLE 1:** Number of practices and patients used for the EMR analysis.

Specialty	Number of practices^a^	Number of physicians^a^	Number of patients^a^ within analysis time span	Physician^a^ per practice^a^	Patients^a^ per practice^a^	Projection factor yearly
GP	1032	1283	2,787,699	1.2	2701	30.7
Pulmonology	40	53	165,873	1.3	4147	16.1
ENT	145	180	1,741,032	1.2	12,007	19.4

Abbreviations: ENT, ear, nose, and throat specialist; GP, general practitioner.

^a^ No personal data but exclusively anonymous information (according to applicable, valid data protection laws).

### 2.4 Parenteral Depot Corticosteroids and Stratifications

Prescriptions of injectable corticosteroids (ATC H02A1) were analyzed and further classified by their New Form Code (NFC) Classification. Only parenteral depot corticosteroids as retard preparations (NFC: GMA, GMY, GNA, GPA) from GPs, ENTs, and pulmonologists were included in the main analysis (PDCs). The number of parenteral non‐depot corticosteroids (NFC: FMA, FMB, FMC, FPB) is listed in the Supporting Information S1; Tables S1 and S2. The number of documented H02A1 prescriptions per patient in the study period were analyzed and further stratified by diagnosis, prescriber specialty, Global Initiative for Asthma (GINA) step (only asthma and LRx data), ASHIP region and age group (both only in LRx data) as well as diagnosis of selected comorbidities (only EMR data).

### 2.5 Human Ethics Statement

As previously described [19, 20, 21], both databases include only anonymized data reported on aggregated level in full compliance with applicable data protection regulations. Under German law, secondary analyses of anonymized data do not require ethics approval or informed consent.

## 3 Results

**FIGURE 1:**
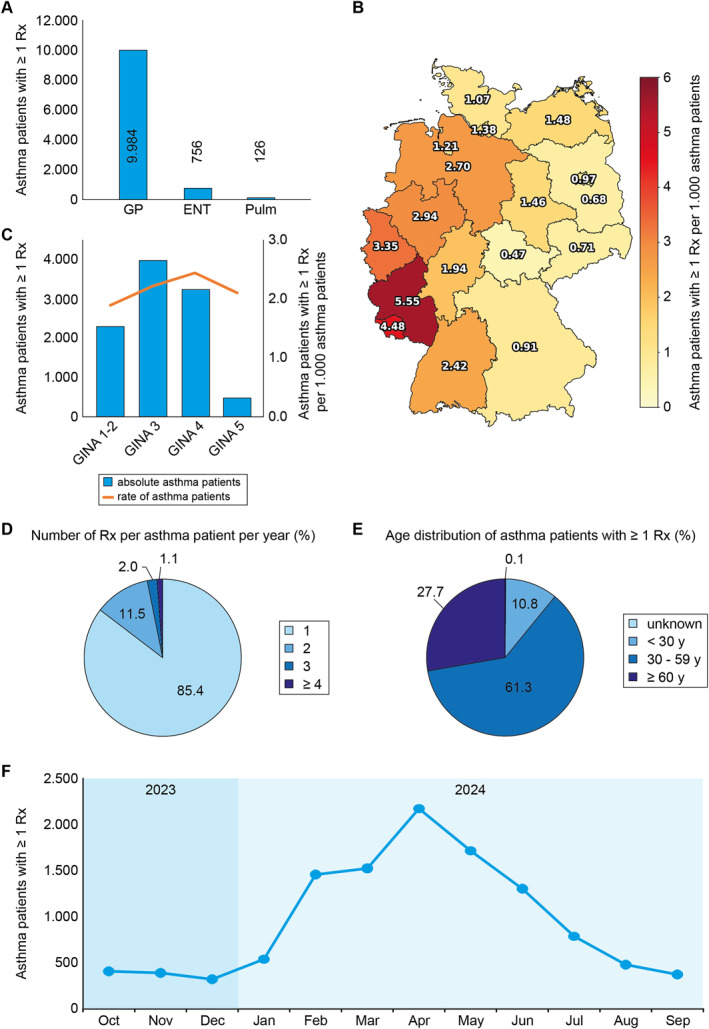
Prescription and regional distribution of parenteral depot steroids use in asthma patients in Germany (prescription data). (A) Number of asthma patients in Germany with at least one prescription of PDCs, stratified by prescriber specialty. (B) Regional data on the number of asthma patients with at least one prescription of PDCs per 1000 asthma patients across 17 ASHIP regions in Germany. (C) Number and rate of asthma patients in Germany with one or more prescriptions stratified by GINA steps. (D) Distribution of asthma patients with at least one prescription of PDCs per year, stratified by the number of prescriptions per year and shown as a percentage of all asthma patients who received PDCs. (E) Age distribution of asthma patients with one or more prescriptions of PDCs. (F) asthma patients with PDC prescriptions per month. (A–F) Analysis period from October 2023 to September 2024 (B–F). Only prescriptions of PDCs from GPs were analyzed. *Data Source:* IQVIA LRx data. ENT, Ear, Nose, and Throat specialist; GINA, Global Initiative for Asthma; GP, General Practitioner; LRx, longitudinal prescription data; Pulm, Pulmonologist; Rx, prescription.

Based on LRx data collected from October 2023 to September 2024, we analyzed the number of asthma patients receiving at least one prescription for PDCs (Figure 1A). We identified 9984 asthma patients with at least one prescription for PDCs from a GP, whereas only 756 and 126 asthma patients received PDCs from an ENT or pulmonologist, respectively. This corresponds to a rate of 2.4 patients with PDC prescriptions per 1000 asthma patients. Regional analysis across 17 ASHIP regions for the share of patients with at least one prescription of PDCs showed substantial differences (Figure 1B). Regions in the western part of Germany exhibited higher shares of PDC use than eastern or south‐eastern regions. Rhineland‐Palatinate and Saarland had the highest rates at 5.55 and 4.48 per 1000 asthma patients, respectively.

PDC use occurred across all GINA steps (Figure 1C). Although the highest absolute patient numbers were assigned to GINA steps 3 and 4, the relative prescription rates were comparable between all GINA steps, ranging from 1.9 to 2.5 per 1000 patients. Since PDCs are often used to treat seasonal allergic symptoms, we analyzed the number of PDC prescriptions per year and per patient (Figure 1D). The vast majority (85.4%) of asthma patients with PDCs received one prescription per year, with 11.5% receiving two. Regarding age distribution, 10.8% of asthma patients with PDC prescription were under the age of 30, nearly two‐thirds (61.39%) were between 30 and 59 years old and 27.7% were 60 years or older (Figure 1E). The number of asthma patients with PDC prescriptions was comparatively low from August to January, between 324 and 543 patients per month. Patient numbers started rising in February, reaching 1467 patients. The number of patients peaked in April with 2186 patients and gradually declined until August (Figure 1F).

**FIGURE 2:**
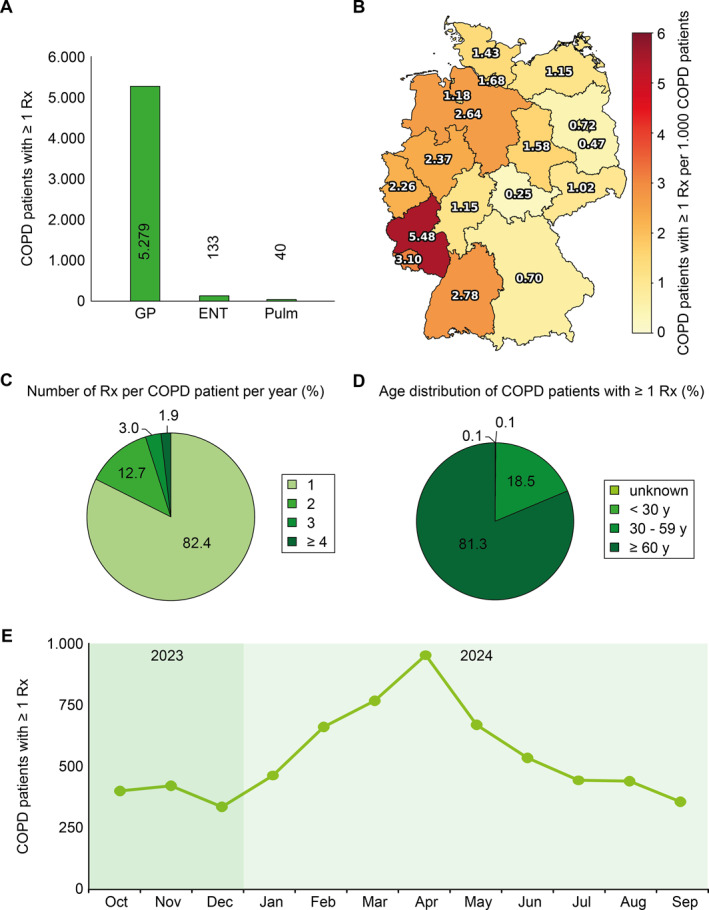
Prescription and regional distribution of parenteral depot steroids use in COPD patients in Germany (prescription data). (A) Number of COPD patients in Germany with at least one prescription of PDCs, stratified by prescriber specialty. (B) Regional data on the number of COPD patients with at least one prescription of PDCs per 1000 COPD patients across 17 ASHIP regions in Germany. (C) Distribution of COPD patients with at least one prescription of PDCs per year, stratified by the number of prescriptions per year and shown as a percentage of all COPD patients who received PDCs. (D) Age distribution of COPD patients with one or more prescriptions of PDCs. (E) COPD patients with PDC prescriptions per month. (A–E) Analysis period from October 2023 to September 2024 (B–E). Only prescriptions of PDCs from GPs were analyzed. *Data Source:* IQVIA LRx data. COPD, chronic obstructive pulmonary disease; ENT, Ear, Nose, and Throat specialist; GP, General Practitioner; LRx, longitudinal prescription data; Pulm, Pulmonologist; Rx, prescription.

Among COPD patients, PDC use followed a similar pattern. In terms of absolute patient numbers, 5279 COPD patients received PDCs from a GP, compared to 133 and 40 patients treated by ENTs or pulmonologists, respectively (Figure 2A). Overall, this represents a rate of 2.0 patients with PDC prescriptions per 1000 COPD patients. Again, the regional prescription pattern parallels the asthma cohort with relatively high rates in the western ASHIP regions (Figure 2B). 82.4% of COPD patients with PDC prescriptions received only one prescription per year and 12.7% received two (Figure 2C). Over 81.3% of COPD patients with PDC prescription were 60 years and older (Figure 2D). The number of COPD patients with PDC prescriptions peaked in April, with 946 patients, and was relatively low, from July to January, ranging from 324 to 433 patients per month (Figure 2E).

**FIGURE 3:**
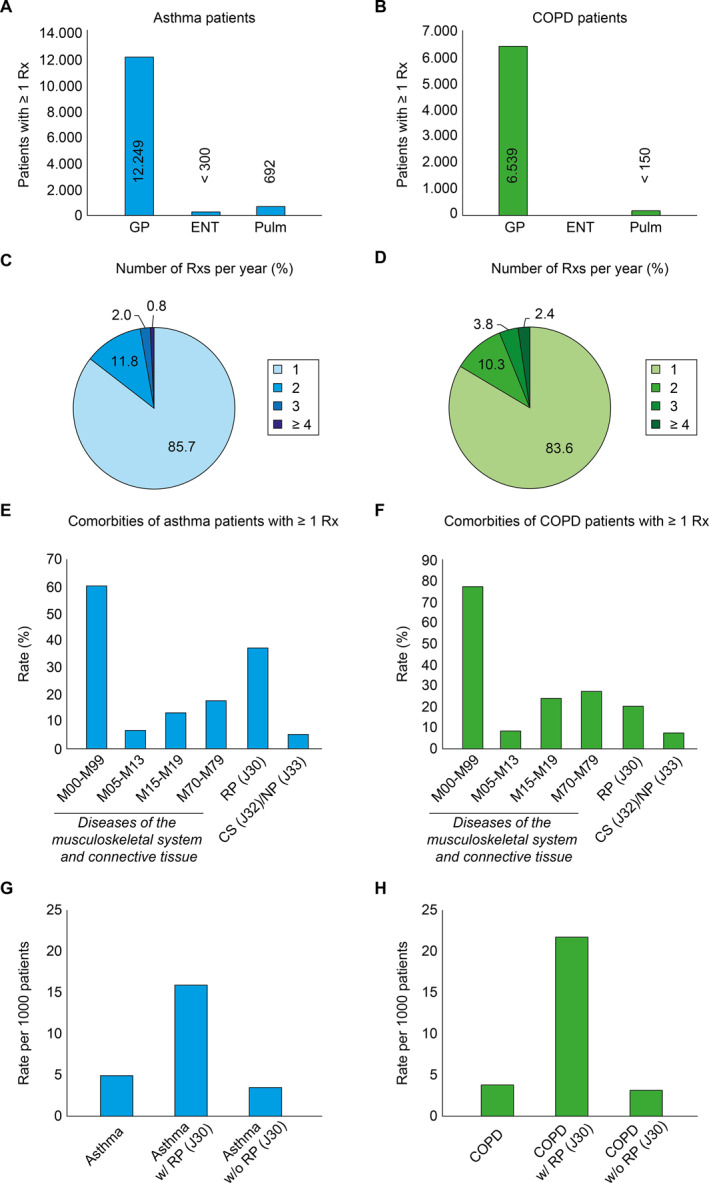
Prescription rate and comorbidities of asthma and COPD patients with parenteral depot corticosteroids (EMR data). (A, B) Projected number of patients with asthma (J45, A) and COPD (J44, B) who received at least one prescription of PDCs during the analysis period. (C, D) Distribution of asthma (C) and COPD (D) patients with at least one prescription of PDCs per year, stratified by the number of prescriptions per year and shown as a percentage of all asthma or COPD patients who received PDCs. (E, F) Rate of selected comorbidities among asthma (E) and COPD (F) patients with at least one prescription of PDCs. (G, H) Rate of asthma (G) and COPD (H) patients with at least one prescription of PDCs with or without comorbid rhinopathy (J30). (A–H) Analysis period from October 2023 to September 2024. Projected EMR data. (C–H) Only prescriptions of PDCs from GPs were analyzed. *Data Source:* IQVIA Disease Analyzer. COPD, chronic obstructive pulmonary disease; CS, chronic sinusitis; ENT, Ear, Nose, and Throat specialist; GP, General Practitioner; ICD‐Codes: M00‐M99, Diseases of the musculoskeletal system and connective tissue; J30, Vasomotor and allergic rhinopathy; J32, Chronic sinusitis; J33, Nasal polyp; M05‐M13, Inflammatory polyarthropathies (excl. arthropathies in diseases classified elsewhere); M15‐M19, Osteoarthritis; M70‐M79, Other diseases of the soft tissue; NP, nasal polyps; PDC, parenteral depot corticosteroids; Pulm, Pulmonologist; RP, vasomotor and allergic rhinopathy (J30); Rx, prescription.

To validate results from the prescription data and provide complementary clinical details regarding comorbidities, we further analyzed anonymized EMR. The projected EMR data confirmed PDC use by GPs with 12,249 asthma patients (Figure 3A) and 6539 COPD patients (Figure 3B) receiving at least one prescription for PDCs in the analysis period. Consistent with LRx data, prescriptions by specialists were infrequent. Similar to the LRx data, most patients in each group received a single PDC prescription per year (Asthma: 85.7%, COPD: 83.6%; Figure 3C, D).

Since PDCs can also be used for treatment of certain diseases of the musculoskeletal system such as arthritis, we analyzed the rate of comorbidities. 60.4% of asthma and 77% of COPD patients had comorbid diseases of the musculoskeletal system and connective tissue (ICD codes M00‐M99; Figure 3E). A more granular analysis on comorbidities of asthma patients with PDC prescriptions revealed that 6.8% were diagnosed with inflammatory polyarthropathies (M05‐M13), 13.3% had osteoarthritis (M15‐M19) and 17.8% had other diseases of the soft tissue (M70‐M79). More than one third (37.3%) had vasomotor and allergic rhinitis (J30) and 5.3% had chronic sinusitis or nasal polyps (J32/J33). For COPD patients with PDCs, 77% had musculoskeletal comorbidities (M00‐M99). Here, 8.5% had comorbidities under M05‐M13, 23.9% had M15‐M19 and 27.2% had M70‐M79 (Figure 3F). The rates of comorbidities classified under J30 and J32/J33 were 20.2% and 7.5%, respectively. Since PDCs are often used to treat seasonal allergic symptoms, we compared the prescription rates of PDCs for asthma and COPD patients with or without rhinopathy (J30), including allergic rhinitis (Figure 3G,H). For asthma, the prescription rate was 14.6 per 1000 patients for patients with comorbid J30 whereas the prescription rate without J30 was only 3.2 per 1000 patients. Similarly, the rate for COPD with J30 was 20.0 per 1000 patients and only 2.9 for patients without J30.

**FIGURE 4:**
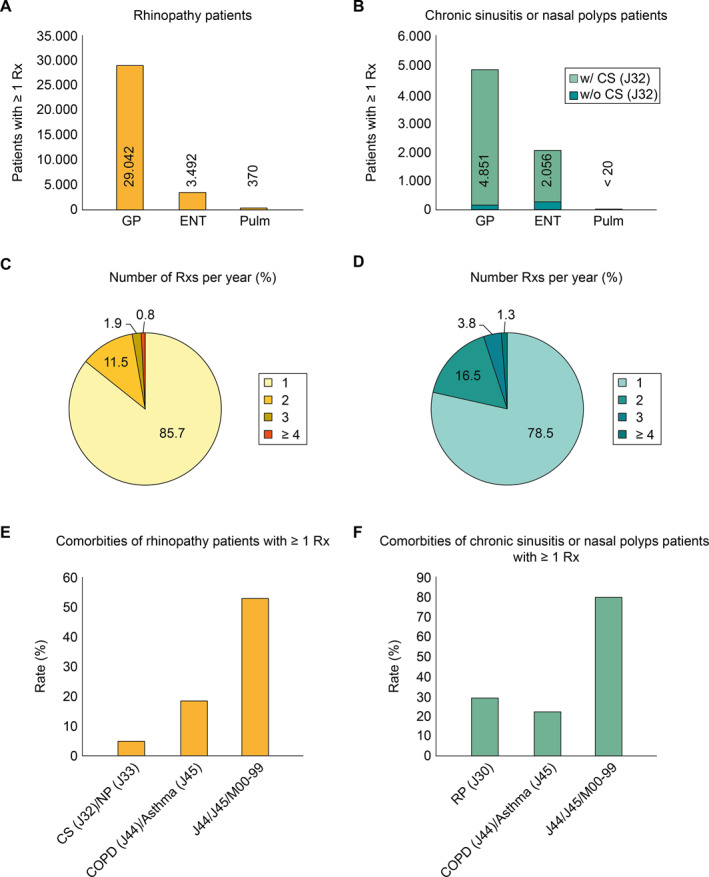
Prescription rate and comorbidities of patients with vasomotor and allergic rhinopathy, chronic sinusitis, or nasal polyps receiving parenteral depot corticosteroids (EMR data). (A, B) Projected number of patients with vasomotor and allergic rhinopathy (J30, A) and chronic sinusitis or nasal polyps (J32/J33, B) who received at least one prescription of PDCs during the analysis period. (C, D) Distribution of J30 (C) and J32/J33 (D) patients with at least one prescription of PDCs per year, stratified by the number of prescriptions per year and shown as a percentage of all J30 and J32/J33 patients who received PDCs. (E, F) Rate of selected comorbidities among J30 (E) and J32/J33 (F) patients with at least one prescription of PDCs. (A–F) Analysis period from October 2023 to September 2024. Projected EMR data. (C–F) Only prescriptions of PDCs from GPs were analyzed. *Data Source*: IQVIA Disease Analyzer. COPD, chronic obstructive pulmonary disease; CS, chronic sinusitis; ENT, Ear, Nose, and Throat specialist; GP, General Practitioner; ICD‐Codes: M00‐M99, Diseases of the musculoskeletal system and connective tissue; J30, vasomotor and allergic rhinopathy; J32, Chronic sinusitis; J33, Nasal polyp; J44: COPD; J45: asthma; NP, nasal polyps; PDC, parenteral depot corticosteroids; Pulm, Pulmonologist; RP, vasomotor and allergic rhinopathy (J30); Rx, prescription.

Patients with vasomotor and allergic rhinopathy represented the largest PDC‐treated cohort (Figure 4A). Here, 29,042 patients received PDC prescriptions from a GP, as well as 3492 patients from an ENT and 370 patients from a pulmonologist. For chronic sinusitis (J32) and/or nasal polyps (J33), 4851 patients treated by GPs received at least one prescription for PDCs as well as 2056 patients treated by ENTs and fewer than 20 patients treated by pulmonologists (Figure 4B). Nearly all these patients had chronic sinusitis (J32) with only 3.2% at GPs and 5.6% at ENTs not having chronic sinusitis and only nasal polyps (J33). Most J30 patients treated at GPs received one prescription in the analysis period (85.7%), and 11.5% received two (Figure 4C). Among patients with J32/J33 code and prescriptions for PDCs, 78.5% received a single prescription and 16.5% received two (Figure 4D).

Regarding comorbidities of J30 patients, 4.9% of the patients treated by GPs had a J32/J33 comorbidity, 18.4% had asthma and/or COPD and 52.9% had either asthma, COPD or a musculoskeletal comorbidity (J45, J44 or M00‐M99; Figure 4E). 29.1% of patients with J32/J33 code and PDC prescription had rhinopathy (J30), 22.2% had asthma and/or COPD and 79.8% had asthma, COPD or a musculoskeletal comorbidity (J45, J44 or M00‐M99; Figure 4F).

## 4 Discussion

Using complementary prescription and EMR data, we document a continued PDC use in respiratory and allergic disease management in Germany. PDCs are predominantly prescribed by GPs and once yearly, consistent with seasonal symptom management. This is additional information to previous studies showing a relevant number of patients with obstructive airway disease receive long‐term treatment with systemic steroids [23, 24, 25]. These findings raise important questions regarding the implementation of guideline‐directed care. The regional distribution is notable as western regions in Germany show substantially higher PDC prescribing rates than eastern or south‐eastern regions. This profile mirrors the documented regional distribution for asthma‐related OCS prescriptions in Germany, suggesting shared, region‐specific prescribing habits and care structures [19, 26]. These similarities indicate that structural and systemic factors such as region‐specific practice norms, local continuing‐education environments, and care structures could be the main drivers that shape the usage of systemic corticosteroids rather than random variation in treatment pattern.

A key finding is the apparent independence of PDC prescribing from asthma severity as determined by GINA treatment steps. Although the largest absolute number of patients falls into GINA steps 3–4, the relative PDC rates are comparable across all GINA steps. In addition, the monthly PDC prescription pattern resembles the pollen count of the most common pollen allergies in Germany starting with hazel and alder in February, birch around April and grass pollen May to July [27, 28]. Combined with the predominant single annual injection frequency and increased prescription rate in patients with J30 comorbidity, this pattern suggests that PDC prescriptions by GPs are primarily used for seasonal allergic symptom management rather than asthma‐specific therapeutic interventions or treatment of comorbidities such as arthritis. From a pharmacological perspective, the usage of PDCs is particularly concerning because depot formulations cannot be promptly discontinued in the event of adverse effects.

PDC prescriptions were concentrated in primary care across all conditions. While GPs manage the vast majority of respiratory and allergic conditions, the increasing complexity in therapeutic options and evolving evidence might create knowledge gaps regarding optimal treatment and risks of long‐term treatments with systemic steroids. Several factors may contribute to the continued use of PDCs including (I) perceived convenience of a single injection during pollen season versus daily treatments, (II) the expectation of rapid symptom relief, (III) potential knowledge gaps regarding adverse effects of systemic steroids, (IV) limited experience with modern, guideline‐directed alternative therapies (e.g., intranasal/topical corticosteroids, second‐generation antihistamines, AIT, biologics), and (V) reimbursement structures and tight time schedules that may favor quick, low‐counseling options. Given clear recommendations against PDCs in allergic diseases by guidelines [8] and the documented risk profile including diabetes, osteoporosis, and local atrophy [1, 3, 5], these findings underscore the need for targeted education and stewardship measures, particularly in primary care.

Methodologically, LRx data captures filled prescriptions, offering large sample sizes and timeliness with the limitation of lacking diagnostic coding and direct comorbidity information. The EMR data on the other hand includes lower patient numbers but offers detailed information on comorbidities. The complementary use of both datasets increases confidence in the observed patterns and provides both population‐level and clinical insights. Both datasets confirm the main findings, namely highest PDC use in primary care and predominantly single annual prescriptions. In addition, the EMR analysis reveals an expected comorbidity pattern of rhinopathy, including allergic rhinitis (J30), and diseases of the musculoskeletal system (M00–M99). While the ICD‐10 code J30 encompasses both allergic and vasomotor rhinopathy, the distinct seasonal peak and annual frequency observed in our data align more with an allergic etiology than with vasomotor rhinitis, which typically presents with perennial symptoms. EMR thus serves as a validating source for LRx‐based hypotheses and fills key information gaps such as diagnoses and comorbidities, even though a high projection factor, especially in case of GPs, remains a limitation.

We cannot fully exclude that PDCs were given for treatment of comorbid diseases such as diseases of the musculoskeletal system or skin diseases. Although the rate of diseases of the musculoskeletal system and connective tissue (ICD‐10 code M00‐M99) is relatively high (60% in asthma patients and 77% in COPD patients with PDCs), this diagnosis is very broad and PDCs are only being used for certain subcategories. These include inflammatory polyarthropathies (M05‐M13; 6.8% in the asthma group and 8.5% in the COPD group) or osteoarthritis (M15‐M19; 13.3% in the asthma group and 23.9% in the COPD group). Regarding the high prevalence of musculoskeletal comorbidities observed in our study, it is important to contextualize these findings within epidemiological associations. Patients with asthma (or to a lesser extent also COPD) exhibit a significantly higher risk of developing rheumatoid arthritis [29, 30]. However, the presence of these comorbidities does not necessarily implicate them as the primary indication for PDC use. Similar to respiratory guidelines, current treatment strategies for rheumatic diseases no longer consider PDCs as ‘state of the art’ therapies and if necessary, should only be used as a bridging strategy [31]. When systemic corticosteroids are required, oral tapering is typically recommended. Consequently, the observed treatment pattern of single and seasonal PDC injections is inconsistent with the management of chronic musculoskeletal or rheumatic conditions, which typically require continuous therapy or treatment of irregular flares. Instead, this distinct prescription pattern rather supports the hypothesis that the observed PDC prescriptions by GPs are primarily driven by seasonal allergic symptom control. An additional limitation of this study is the lack of data on disease control, quality of life, or adverse event rates, limiting our ability to quantify the clinical impact of the observed prescription pattern. Due to the cross‐sectional nature of this study, we cannot assess the trends on the development of PDC prescription over time. Future longitudinal studies should examine whether educational activities or policy changes alter PDC use, and could incorporate patient‐journey elements, allergen exposure, concomitant use of guideline‐directed therapies such as intranasal corticosteroids and antihistamines, as well as outcome measures such as exacerbations, healthcare contacts, and adverse events.

In the context of guidelines, this pattern warrants critical appraisal. For allergic rhinitis and related conditions, systemic steroids are recommended only in narrowly defined situations and not as first‐line therapy with depot injections explicitly not being considered as a routine option [8, 9, 10]. For asthma, OCS is indicated for exacerbations and, as maintenance, only under well‐defined circumstances where other treatment options failed to restore symptom control [11]. In modern therapeutic concepts the goal of asthma management is remission, which should be reached by using disease‐modifying anti‐asthmatic drugs (DMAADs) such as topic corticosteroids, biologics and AIT but not with systemic corticosteroids [32]. The seasonal and GINA step‐independent PDC pattern observed in this study is not aligned with guideline recommendations. Practical implications include medical education programs that emphasize PDC risks and the effectiveness of modern anti‐allergic and topical therapies, topical therapies, AIT and biologics. Quality improvement programs targeting high‐prescribing areas could include peer comparison feedback and practice‐based learning collaboratives that are tailored to address region‐specific needs. In this regards, multidisciplinary team approaches should be followed to increase the awareness of PDC risks and to optimize management of patients in a holistic way [33]. Professional societies and regional physician associations should scrutinize the local PDC prescription habits and address specific regional factors contributing to high PDC utilization. Finally, policy approaches should be considered such as prior authorization requirements for PDC prescriptions, mandatory specialist consultation or preferential reimbursement for evidence‐ and guideline‐directed therapies.

## 5 Conclusion

Our data demonstrate a continued, presumably allergy‐driven PDC use in primary care with substantial regional variation. In light of clear guideline positions and the unfavorable risk–benefit profile of PDCs, the findings support targeted measures to reduce PDC prescribing in favor of safer and more effective alternatives. The scale of guideline‐discordant prescribing identified underscores the need for systematic interventions addressing both provider education and healthcare system factors. It further highlights the need for effective interventions to minimize PDC use, in order to meaningfully improve patient outcomes and quality of care.

## Author Contributions

**Christian Taube:** writing – original draft, writing – review and editing. **Timm Greulich:** writing – review and editing, **Sebastian Böing:** writing – review and editing. **Oliver Pfaar:** writing – review and editing. **Martin Wagenmann:** writing – review and editing. **Sven Becker:** writing – review and editing. **Mathias Sulk:** writing – review and editing. **Niklas J. Gerkau:** writing – review and editing, visualization, writing – original draft, formal analysis, investigation. **Jan Claudius Schwitalla:** writing – original draft, writing – review and editing, investigation, conceptualization, formal analysis, supervision, project administration, visualization, validation, data curation, funding acquisition. **Tim Vierbuchen:** writing – review and editing, writing – original draft, conceptualization, investigation, formal analysis, supervision, project administration, visualization, validation, data curation.

## Conflicts of Interest

This study was supported by AstraZeneca GmbH, Hamburg, Germany. CT received honoraria for lectures and/or consulting activities from AstraZeneca, BerlinChemie, Chiesi, GSK, Novartis, Sanofi; travel support from AstraZeneca, Sanofi. TG received honoraria for lectures and/or consulting activities from AstraZeneca, BerlinChemie, Boehringer‐Ingelheim, Chiesi, CSL‐Behring, GSK, Grifols, Insmed, Novartis, Orion Pharma, Sanofi; travel support from AstraZeneca, Chiesi, Grifols; and research support from Grifols. SeBö received honoraria for lectures, consulting activities and/or travel support from ALK, AstraZeneca, Berlin‐Chemie, Boehringer Ingelheim, Chiesi, GlaxoSmithKline, HAL, Löwenstein Medical, Novartis, ResMed, Sanofi, Stallergenes Greer.OP received honoraria for lectures, consulting activities, research grants and/or travel support from AEDA, Alfried Krupp Krankenhaus, ALK‐Abelló, Allergopharma, Almirall, Altamira Therapeutics, ASIT Biotech, AstraZeneca, Bencard Allergie GmbH/Allergy Therapeutics, Blueprint, Breazy Health, Cliantha, Deutsche AllergieLiga e.V., Deutsche Forschungsgemeinschaft, Dustri‐Verlag, ECM Expro&Conference Management GmBH, Forum für Medizinische Fortbildung, Georg‐Thieme‐Verlag, GSK, HAL Allergy Holding B.V./HAL Allergie GmbH, Inmunotek, Ingress Health, Institut für Disease Management, IQVIA Commercial, Japanese Society of Allergology, Königlich Dänisches Generalkonsulat, Laboratorios LETI/LETI Pharma, Lilly, Lofarma, Medizinische Hochschule Hannover, med update europe GmbH, Meinhardt Congress GmbH, Novartis, Paul‐Ehrlich‐Institut, Paul‐Martini‐Stiftung, PneumoLive, Pohl‐Boskamp, Procter & Gamble, Red Maple Trials Inc., Regeneron, RG Aerztefortbildung, ROXALL Medizin, Sanofi Aventis, Sanofi Genzyme, Springer Publisher, Stallergenes Greer, streamedup! GmbH, Technical University Dresden, John Wiley & sons publishers, Wort & Bild Verlag, Verlag ME; all outside the submitted work, Oliver Pfaar is Vice President of the European Academy of Allergy and Clinical Immunology (EAACI), a member of EAACI Excom as well as a member of the external board of directors of the German Society of Allergy and Clinical Immunology (DGAKI); coordinator, main‐ or co‐author of different position papers and guidelines in rhinology, allergology and allergen‐immunotherapy; and he is Editor‐in‐Chief of Clinical Translational Allergy and Associate Editor of Allergy. MW received honoraria for lectures, consulting activities, and/or research grants from Allergopharma, ALK‐Abelló, AstraZeneca, CSL‐Behring, EU, Genzyme, GSK, HAL Allergie, Infectopharm, LETI Pharma, MSD, NeilMed, Novartis, Regeneron, Sanofi, Stallergenes, Takeda. SvBe received honoraria for lectures, consulting activities, and/or research grants from ALK‐Abelló, Allergopharma, Allergy Therapeutics, Ambu, AstraZeneca, Altamira AG, Auris medical, Bencard Allergie, GSK, HAL Allergie, MSD, Mylan, Novartis, Sanofi‐Genzyme, Stryker und Viatris. SvBe is vice president of Ärzteverband Deutscher Allergologen e. V. (AeDA), chair of Arbeitsgruppe klinische Immunologie, Allergologie und Umweltmedizin der deutschen Gesellschaft für Hals‐Nasen‐Ohren‐Heilkunde and vice president of the German CRS registry. MS received honoraria for lectures and/or consulting activities from Ärzteverband deutscher Allergologen, Ärztekammer Westfalen Lippe, AstraZeneca, Bencard, BioCryst, Blueprint Medicines, CSL Behring, Kalvista, HAL Allergie, Healthcare Deutschland, LEO Pharma, Monasterium Laboratory, Novartis, RG Gesellschaft für Information und Organisation, Sanofi, Stallergenes, Takeda, Unna Akademie. NJG, JCS, and TV are employees of AstraZeneca and may hold shares or share options in the company.

## Supporting information


Supporting Information S1

## Data Availability

The data that supports the findings of this study are available in the supplementary material of this article or available from the corresponding author upon reasonable request.
